# Supplementary material for: A three years antimicrobials consumption in Ethiopia from 2017 to 2019: A cross- sectional study
Source: PLoS One. 2023 Apr 6;18(4):e0284038. doi: 10.1371/journal.pone.0284038 (PMC10079031; doi:10.1371/journal.pone.0284038)
Supplement: Attachment — Submitted filename: Response Letter to the Editor and Reviewers.docx [file pone.0284038.s001.docx]

**Date: March 7, 2023**

**Response Letter to the Editor and Reviewers**

We appreciate the editor and both reviewers’ time, comments and suggestions provided for our article entitled “**A three years antimicrobials consumption in Ethiopia from 2017 to 2019: A cross- sectional study: Manuscript ID=** **PONE-D-22-35443**”. Reviewer comments motivated thorough revision of the manuscript, which is being resubmitted along with a point-by-point response to address all reviewer concerns (Table below). All modifications are shown in the revised manuscript attached with file name of “Manuscript” for manuscript without track change and “Revised Manuscript with track changes” for the file highlighted with changes. Thank you for the valuable comments and we hope the editor and reviewers will be satisfied with our responses.

**Sincerely,**

**Atalay Mulu Fentie, on behalf of all authors**

| **S.No** | | **Query by** | **Response** | | |
| --- | --- | --- | --- | --- | --- |
| 1. | | **Editor** |  | | |
| 1. 1.1. | | Please ensure that your manuscript meets PLOS ONE's style requirements, including those for file naming. | All authors have thoroughly reviewed the manuscript and made changes as required to meet all of the PLOS ONE style requirements. In addition, all authors addressed identified grammatical and typographic errors (see the revised manuscript with track changes). | | |
| 2. 1.2. | | In your Methods section, please confirm that all data sources you used were publicly available and anonymized. If this is not the case, please provide information on what permissions you were granted to access these data. | Thank you so much. Now, we have included “All data sources used in this study were publicly available and anonymized” under source of data Methods sub-section of the revised manuscript. | | |
| 3. 1.3. | Thank you for stating the following financial disclosure:  "No. The funders had no role in study design, data collection and analysis, decision to publish, or preparation of the manuscript"  At this time, please address the following queries:  a) Please clarify the sources of funding (financial or material support) for your study. List the grants or organizations that supported your study, including funding received from your institution.  b) State what role the funders took in the study. If the funders had no role in your study, please state: “The funders had no role in study design, data collection and analysis, decision to publish, or preparation of the manuscript.”  c) If any authors received a salary from any of your funders, please state which authors and which funders.  d) If you did not receive any funding for this study, please state: “The authors received no specific funding for this work.”  Please include your amended statements within your cover letter; we will change the online submission form on your behalf. | | | Now we have the final disclosure section as per your recommendations and on the revised manuscript it appears as follows:  “This national survey was financially supported by Ethiopian Food and Drug Authority through the annual government budget allocated specifically for the consumption survey. However, the funder had no role in study design, data collection and analysis, decision to publish, or preparation of the manuscript. In connection with this, the authors received no specific funding for this work.” |  |
| 4. 1.4. | | In your Data Availability statement, you have not specified where the minimal data set underlying the results described in your manuscript can be found. | We have amended the data availability statement as follows and included it on the revised manuscript. “Minimum data relevant to this study are anonymized and included in this article”. | | |
| 5. 1.5. | | Please review your reference list to ensure that it is complete and correct. | We have revised the reference section as per PLOS ONE reference writing guideline, and we hereby confirm it is complete and correct. | | |
| **2. 2.** | | **Reviewer 1-** **Dr. Rozafa Koliqi** |  | | |
| 2.1. | | There should be a space between the word after or before it and all the references should be the same, a space before it or not. | Thank you. As per the kind comment, we have thoroughly reviewed the manuscript and amended it accordingly. | | |
| 2.2. | | At the table 4. You have present most commonly consumed antimicrobials by chemical substance group in Ethiopia between 2017-2019. Is there any explanation why is such a big difference at DID between the year 2017 and two other years (2018 and 2019)? | It could be due to the acute watery diarrheal outbreak in Ethiopia in 2017 that leads the consumption of antimicrobials high. If you look at the consumption of Doxycycline, it was significantly higher given doxycycline was recommended on the emergency preparedness and management of acute diarrheal guideline as first line treatment. We have included the same on the discussion section of the manuscript on paragraph 4. | | |
| 2.3. | | A number of similar studies have been published in past by a number of research groups. Moreover, current findings are also not conceptually and technically different from the earlier published studies. Due to the large sample, my recommendation is to the authors that they should emphasize and elaborate the novel findings and speculate what are the contribution of this study to previously published studies. | Thank you for this comment and yes, recently we have developed the AMR prevention and containment national action plan of Ethiopia. In line to this, one of the strategic objective is research and surveillance to support all other intervention strategies and policies in which this study was conducted in accordance with it. Moreover, we have revised the Essential medicine list, standard treatment guideline, health insurance medicine list and community pharmacy list; and categorized antibiotics as per Access, Watch and Reserve (AWaRe). We tried discuss and present the antimicrobial consumption as per AWaRe category that will serve a baseline for further study as well as assess the five years national action plan impact on antimicrobial consumption and AMR. | | |
| 3. | | **Reviewer 2** |  | | |
| 3.1. | | Overall the article is written in a very comprehensive way. Chosen methodology is correct. Using DID gives comparable results and data for further drug utilisation studies. However it should be mentioned that it should be good to have also results that for the most consumed antibiotics in order to create a link with the resistance as well. One other suggestion is also to describe the fluctuations of different classes of antibiotics through the years and if there is supporting evidence in prescriptions of these antibiotics. | Thank you for your suggestion. We tried to highlight the findings of studies conducted in Ethiopia regarding to antimicrobial resistance pattern/issue on the discussion section of the manuscript. Moreover, the reasons for fluctuation of the consumption was also discussed. The only supporting evidence we found is the issue of acute watery diarrhea in 2017 that significantly increased the consumption compared to 2018 and 2019. | | |
